# A Distributed Localization Method for Wireless Sensor Networks Based on Anchor Node Optimal Selection and Particle Filter

**DOI:** 10.3390/s22031003

**Published:** 2022-01-27

**Authors:** Qinghua Luo, Chao Liu, Xiaozhen Yan, Yang Shao, Kexin Yang, Chenxu Wang, Zhiquan Zhou

**Affiliations:** 1 School of Information Science and Engineering, Harbin Institute of Technology, Weihai 264209, China; 20S130383@stu.hit.edu.cn (C.L.); yanxiaozhen@hit.edu.cn (X.Y.); 20S130384@stu.hit.edu.cn (Y.S.); 21S130275@stu.hit.edu.cn (K.Y.); wangchenxu@hit.edu.cn (C.W.); zzq@hitwh.edu.cn (Z.Z.); 2 Shandong Institute of Shipbuilding Technology, Weihai 264209, China; 3 Shandong New Beiyang Information Technology Co., Ltd., Weihai 264209, China

**Keywords:** distributed localization, wireless sensor networks (WSNs), anchor node optimization, particle filter

## Abstract

In wireless sensor networks, due to the significance of the location information of mobile nodes for many applications, location services are the basis of many application scenarios. However, node state and communication uncertainty affect the distance estimation and position calculation of the range-based localization method, which makes it difficult to guarantee the localization accuracy and the system robustness of the distributed localization system. In this paper, we propose a distributed localization method based on anchor nodes selection and particle filter optimization. In this method, we first analyze the uncertainty of error propagation to the least-squares localization method. According to the proportional relation between localization error and uncertainty propagation, anchor nodes are selected optimally in real-time during the movement of mobile nodes. Then we use the ranging and position of the optimally selected anchor nodes to obtain the location information of the mobile nodes. Finally, the particle filter (PF) algorithm is utilized to gain the optimal estimation of the localization results. The experimental evaluation results verified that the proposed method effectively improves the localization accuracy and the robustness of the distributed system.

## 1. Introduction

Wireless sensor networks (WSNs) are applied in numerous application scenarios [1,2,3], such as environmental monitoring, smart cities, disaster relief, and asset tracking, which all require precise location services of nodes, especially moving object tracking. As universal methods, the Global Positioning System (GPS) and the BeiDou Navigation Satellite System (BDS) provide location services. However, their positioning accuracy is reduced significantly in buildings, indoors, or canyons [4,5]. That makes it difficult to obtain reliable positioning information.

Wireless sensor networks comprise anchor nodes with known locations and mobile nodes with unknown locations. In recent years, scholars have proposed many WSN location algorithms to obtain the accurate location estimation of sensor nodes. For example, localization algorithms can be classified into range-based and range-free localization methods depending on ranging [6]. To calculate the position of the moving nodes with the absolute distance or angle information between the nodes, range-based localization methods adopt different algorithms, such as trilateration, triangulation, least squares, and maximum likelihood estimation [7]. At the same time, the range-free localization algorithms make use of some information such as network connectivity and estimated distance between nodes to realize positioning, which includes the distance vector-hop (DV-hop) positioning algorithm, the centroid algorithm, the approximate point-in-triangulation test (APIT) positioning algorithm, the convex optimization positioning algorithm [8,9], etc. In addition, according to the information of the WSN nodes processed in different sites, the localization algorithms can be divided into centralized localization and distributed localization algorithms [10]. To calculate the position, the centralized localization methods transmit the information of mobile nodes to a central node, while the distributed localization methods utilize the information exchange and coordination between the nodes [11]. In multiple mobile nodes localization scenarios, the distributed localization methods possess higher efficiency and accuracy than those of the centralized localization methods. To meet the requirement of more practical applications, we have studied the distributed localization of mobile nodes based on the range-based localization method to improve the accuracy and efficiency of WSN localization.

The positioning accuracy of the mobile nodes depends on both the distance estimation of anchor nodes and the position calculation methods. Due to the complex environmental factors during wireless signal propagation such as environmental interference, reflection, refraction, multi-path, and none line of sight (NLOS) transmission [5,12], there are different degrees of error in distance estimation. At the same time, the inevitable defects of wireless sensor networks enlarge the input error, where the anchor node may have the problems of communication uncertainty [13], limited processing capacities, and insufficient power supply [14] of the sensor node. Therefore, it is necessary to select reliable anchor nodes for distance estimation and positioning calculations during the process of the distributed localization. In addition, because of the nonlinear motion of the located mobile node, the positioning accuracy is affected by the positioning calculation method as well. To improve the dynamic positioning accuracy in the case of the ranging information changing constantly, a filtering algorithm needs to be used to optimize the initial positioning results after selecting anchor nodes for the range-based positioning method. 

In this paper, we comprehensively analyze the error propagation mechanism of the distributed mobile nodes through WSN to improve the localization accuracy and the robustness of the system at first. Then we adopt the minimum standard deviation optimization (MSDO) criterion and the minimum error propagation optimization (MEPO) criterion to select reliable anchor nodes. Finally, the initial positioning results are optimized through the PF algorithm after anchor node selection to obtain the final accurate position. Combining the MSDO and MEPO criteria with the particle filter algorithm, we propose the distributed localization method based on anchor node optimal selection and particle filter (MSDO-PF and MEPO-PF).

## 2. Related Works

There are some factors limiting wireless sensor networks, such as the processing capacity, storage memory, energy consumption, fixed deployment, and outdoor harsh conditions. These affect the reliability of the network and node localization accuracy seriously. Aiming at the uncertainty of WSN that profoundly affects the network reliability, many scholars have put forward different methods to improve location performance.

Considering the errors of anchor nodes both in range-based and range-free localization methods, authors in [15] presented a sequential greedy optimization algorithm, which is more suitable for distributed optimization than the classical nonlinear Gauss-Seidel algorithm. Authors in [16] calculated the similarity between nodes according to the location information and hops of anchor nodes, while using the K most similar anchor nodes to calculate the coordinates of unknown nodes. Then it proposed a distributed location algorithm based on K nearest neighbor classification to further improve the positioning accuracy of a traditional K-Nearest Neighbor (KNN) algorithm, which determines the similarity to the node location information. Aiming at understanding the way that the redundancy and the node deployment affect the network reliability, reference [5] analyzed the design and implementation of a wireless sensor network for low-power and low-cost applications while calculating its reliability based on the real environmental conditions and arrangement of the nodes deployed in the field. Authors in [17] proposed an uncertain dynamic data stream clustering algorithm based on the interval numbers, which improved the clustering accuracy by 61%. The research results verified the feasibility and effectiveness of the interval number uncertainty processing method. Similarly, authors in [18] proposed two combinatorial optimization problems and two heuristic algorithms. DV-Hop is a popular localization technology; authors in [19] proposed a centroid DV-hop localization with selected anchors and inverse distance weighting schemes (SIC-DV-Hop), an algorithm that can significantly improve performance and cost less as a network resource. However, it is necessary to further study the system for managing uncertainty, which includes the propagation of various uncertain factors in the system and the comprehensive evaluation of system output uncertainty.

In addition, when performing the location of nonlinear mobile node, both the position of the nodes and the ranging information from the anchor nodes to the mobile node change constantly. To solve the communication uncertainty caused by the failure of sensor nodes and mobile targets including packet loss, data disorder, and time to delay, authors in [13] proposed an adaptive fading factor to compensate for the inconsistency and error of the estimation. In [20], the Kalman filter (KF) method was used to perform positioning. However, the state and measurement equations were assumed to be linear in this method, which was inconsistent with the actual situation. Considering the limitation of the KF algorithm only dealing with linear systems, an Extended Kalman filter (EKF) algorithm based on Taylor series expansion was proposed, which was applied to the dynamic positioning of the nonlinear system [21]. After that, the Unscented Kalman filter (UKF) based on deterministic sampling was proposed to perform the positioning of the nonlinear system [22,23], which was better than the EKF algorithm according to the experimental results. Under the circumstances of the non-Gaussian and nonlinear systems requiring dynamic positioning and navigation, reference [24] adopted the PF algorithm based on Monte Carlo sampling, in which the combination of multiple dynamic positioning methods was utilized to perform dynamic positioning in a complex environment.

In the above methods, there are still some limitations in improving the localization accuracy. Considering the uncertainty of anchor node position, the localization system has not been improved universally due to the lack of analysis after error propagation. In this paper, considering the uncertainty of error propagation caused by some negative factors, we adopt the MSDO and MEPO methods, after which we propose the distributed localization method based on the MSDO-PF and MEPO-PF algorithms to optimize the positioning results.

In this paper, we make the following contributions:

- Based on the uncertainty analyzing of the error propagation in the least-squares localization method, we find that localization error is correlated positively with both the statistic standard deviation of distance estimation and the product of distance statistic standard deviation and distance;
- According to the minimum standard deviation and the minimum error propagation factor, the anchor node is optimized in real-time during the process of node movement, after which the distance measurement and position information about the optimized anchor nodes is brought into the least-squares localization method to obtain the initial position information about the mobile node;
- To get more accurate positioning information and improve the system’s robustness, we treat the position information of the mobile nodes as the initial position estimation value of the PF algorithm. Simulation results show that the MSDO-PF and MEPO-PF methods can effectively improve the positioning accuracy of distributed mobile nodes and the system’s robustness.

## 3. Distributed Localization Method for Wireless Sensor Networks Based on Anchor Node Optimal Selection and Particle Filter

### 3.1. System Structure

**Figure 1:**
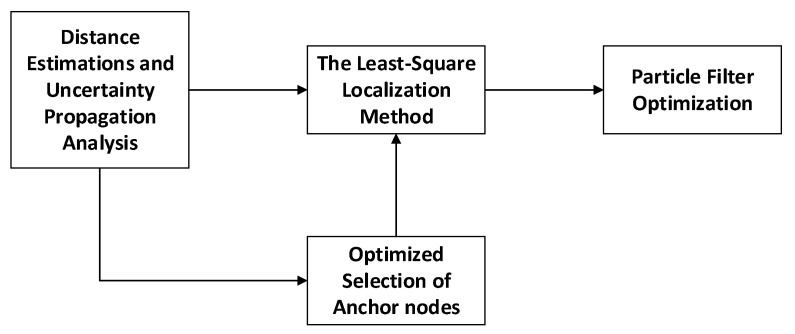
The framework of MSDO-PF and MEPO-PF localization method.

We illustrate the framework of the distributed localization method based on anchor node selection and particle filter optimization in Figure 1. It comprises the following sub-modules: a distance estimation and uncertainty propagation analysis module, an optimized selection of anchor nodes module, a least-squares localization module, and a particle filter optimization module.

The implementation steps of each part are as follows:

- Distance estimation and uncertainty propagation analysis: In the wireless sensor network system, we measure the distance between the mobile node and each anchor node repeatedly. Then we statistically calculate to obtain the distance estimation result. We calculate the statistical standard deviation, representing the quality of the distance estimation, and the product of the distance estimation and the statistical standard deviation (defined as the error propagation factor). According to the minimum standard deviation criteria and the minimum error propagation factor criteria, we propose the MSDO and MEPO methods to select the anchor nodes optimally;
- Optimal selection of anchor nodes: According to MSDO and MEPO methods, we sort the anchor nodes and obtain the corresponding indexes. We select a different number of anchor nodes in turn for different localization algorithms. In this paper, we choose the first five anchor nodes into the least-squares localization method;
- Least-squares localization: Based on the selected anchor nodes and their corresponding distance estimation result, we can obtain an accurate preliminary localization result through the least-squares criterion;
- Particle filter optimization: To ensure the distributed nonlinear localization system has higher localization accuracy and stronger robustness, we treat the initial location as the input. We utilize the particle filter algorithm to optimize the estimation localization result.

### 3.2. Least-Squares Localization

When analyzing the system structure, we first introduce the least-squares location method, as the quality of the anchor nodes is evaluated for the least-squares localization method. After the anchor nodes are optimized, their coordinates and corresponding distance estimation results are also brought into the least-squares localization method to calculate the initial location of the mobile node.

**Figure 2:**
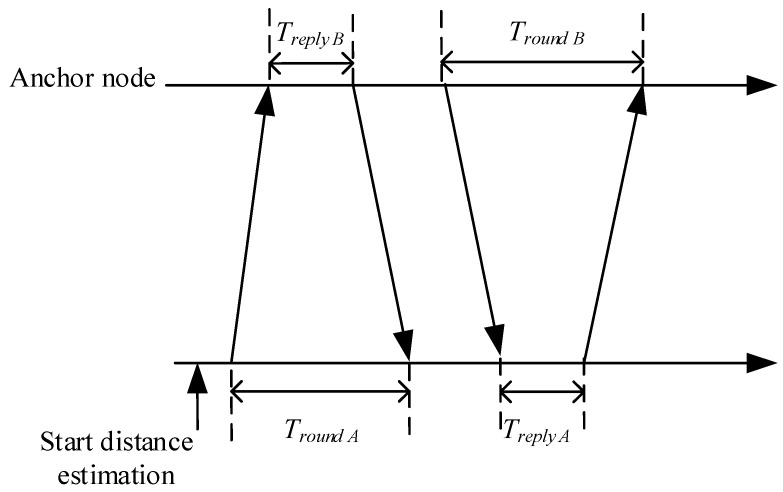
The procedure of DS-TWR distance estimation.

Before the least-squares method positioning, the distance estimation between the anchor node and the mobile node needs to be explained. The distance estimation of the anchor node and the mobile node can use RSSI, AOA, TOA, TDOA, SS-TWR, DS-TWR and other methods. In our method, we use DS-TWR, which is the most widely used distance estimation method. We show the procedure of a DS-TWR distance estimation between an unknown node and an anchor node in Figure 2.

DS-TWR distance estimation method adds another communication based on SS-TWR distance estimation method, and the time of two communications can make up for the error caused by clock offset. The distance estimation between the anchor node and mobile node can be calculated using the Equation (1). In this equation, $T_{round\;A}$, $T_{round\;B}$ denote the propagation delay from one node to another node, and $T_{reply\;A}$, $T_{reply\;B}$ denote the processing delay of the anchor node. v denotes the propagation velocity of radio signal.

(1) $$d=\frac{T_{round,A}\times T_{round,B}-T_{reply,A}\times T_{reply,B}}{T_{round,A}+T_{round,B}+T_{reply,A}+T_{reply,B}}\times v$$

In this paper, to get higher accuracy distance estimation, it is estimated repeated N times, and each measurement is $d_{1n}$, N∈N, 1≤n≤N. For example, for ranging between anchor node $A_{1}$ and an unknown node, the average mean $d_{1}$ is statistically calculated as the distance estimation results, and we adopt the standard deviation $\sigma_{d_{1}}$ as the uncertain information.

(2) $$d_{1}=\frac{1}{N}\sum_{n=1}^{N}d_{1n}$$

(3) $$\begin{array}{ll} \sigma_{d_{1}} & =(\frac{1}{N-1}\sum_{n=1}^{N}(d_{1n}-d_{1}){)}^{1/2}\\ & =(\frac{1}{N-1}\sum_{n=1}^{N}(d_{1n}-\frac{1}{N}\sum_{n=1}^{N}d_{1n}){)}^{1/2} \end{array}$$

**Figure 3:**
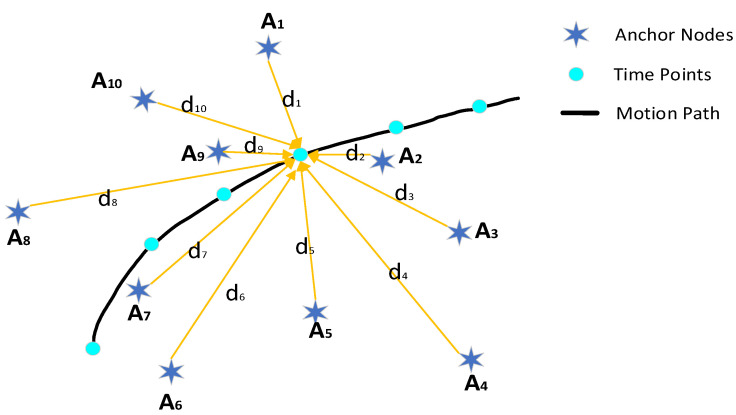
Anchor nodes and mobile nodes’ path.

As shown in Figure 3, we assume that there are *k* known anchor nodes $A_{}=\left\{A_{1},A_{2},\cdots ,A_{i},\cdots A_{k}\right\}$ with corresponding coordinates as follows: $\left(x_{1},y_{1}\right),\left(x_{2},y_{2}\right),\cdots \left(x_{i},y_{i}\right),\cdots \left(x_{k},y_{k}\right)$ (i=1,2,⋯,k), respectively. Suppose the position coordinate of the unknown node is (x,y), the corresponding distance estimated by the anchor nodes are $d=\left\{d_{1},d_{2},\cdots d_{i},\cdots ,d_{k}\right\}$.

The localization equations can be formed as follows:

(4) $$\{\begin{array}{c} \sqrt{{\left(x-x_{1}\right)}^{2}+{\left(y-y_{1}\right)}^{2}}=d_{1}\\ \sqrt{{\left(x-x_{2}\right)}^{2}+{\left(y-y_{2}\right)}^{2}}=d_{2}\\ \vdots \\ \sqrt{{\left(x-x_{k}\right)}^{2}+{\left(y-y_{k}\right)}^{2}}=d_{k} \end{array}$$

In the form of matrix equality, where the matrices *A*, *B* and *X* are defined as follows, respectively:

(5) $$A=\left[\begin{array}{cc} \left(x-x_{1}\right) & \left(y-y_{1}\right)\\ \left(x-x_{2}\right) & \left(y-y_{2}\right)\\ \vdots & \vdots \\ \left(x-x_{k}\right) & \left(y-y_{k}\right) \end{array}\right]$$

(6) $$X=\left[\begin{array}{c} x\\ y \end{array}\right]$$

(7) $$B=\left[\begin{array}{c} x_{k}^{2}-x_{1}^{2}+y_{k}^{2}-y_{1}^{2}+d_{1}^{2}-d_{k}^{2}\\ x_{k}^{2}-x_{2}^{2}+y_{k}^{2}-y_{2}^{2}+d_{2}^{2}-d_{k}^{2}\\ \vdots \\ x_{k}^{2}-x_{k-1}^{2}+y_{k}^{2}-y_{k-1}^{2}+d_{k-1}^{2}-d_{k}^{2} \end{array}\right]$$

According to the principle of the least-squares method, the condition that the unknown node coordinate should satisfy is that the square sum of all measured distance and its corresponding actual distance error is minimum. Equation (4) is derived as linear square difference by the least-squares method, and the form is:

(8) $$\underset{x,y}{\min}{\left\|AX-B\right\|}_{2}$$

So, based on the least-squares criterion, we can obtain the solution for the location equations:

(9) $$X={\left(A^{T}A\right)}^{-1}A^{T}B$$

For the distributed nonlinear mobile positioning system, we locate the moving nodes by the least-squares method based on the distance measurement, the position coordinates of each moving node can be obtained.

### 3.3. Uncertainty Propagation Analysis and Optimal Selection of Anchor Nodes

When calculating the coordinates of moving nodes, one of the variables with uncertainty is the anchor node coordinate $\left(x_{i},y_{i}\right)$(i=1,2,⋯,k), whose size is the sum of the actual value and a neighborhood not less than zero, which is decomposed into $\left(\delta_{x_{i}},\delta_{y_{i}}\right)$ in a rectangular coordinating system; The other uncertainty is the distance estimation $d_{i}$ of the corresponding anchor node to the moving node, and the error is $\delta_{d_{i}}$. The sensitivity coefficients of anchor node coordinate and distance estimation are defined as follows:

(10) $$S_{x_{i}}=\frac{\partial ({\left(A^{T}A\right)}^{-1}A^{T}B)}{\partial x_{i}}$$

(11) $$S_{y_{i}}=\frac{\partial ({\left(A^{T}A\right)}^{-1}A^{T}B)}{\partial y_{i}}$$

(12) $$S_{d_{i}}=\frac{\partial ({\left(A^{T}A\right)}^{-1}A^{T}B)}{\partial d_{i}}\left(i=1,2,\cdots ,k\right)$$

According to the total differentiation formula (TDF), we can obtain the positioning error as the following result:

(13) $$\begin{array}{ll} \left(\begin{array}{c} \delta x\\ \delta y \end{array}\right) & =\sum_{i=1}^{k}\left(S_{x_{i}}\delta_{x_{i}}+S_{y_{i}}\delta_{y_{i}}+S_{d_{i}}\delta_{d_{i}}\right)\\ & =\sum_{i=1}^{k}\left(\frac{\partial ({\left(A^{T}A\right)}^{-1}A^{T}B)}{\partial x_{i}}\delta_{x_{i}}+\frac{\partial ({\left(A^{T}A\right)}^{-1}A^{T}B)}{\partial y_{i}}\delta_{y_{i}}+\frac{\partial ({\left(A^{T}A\right)}^{-1}A^{T}B)}{\partial d_{i}}\delta_{d_{i}}\right) \end{array}$$

When we arrange the site, we can minimize the coordinate error of the anchor node by using relatively accurate calipers to determine the location of the anchor nodes. Therefore, we ignore the coordinate error $\delta_{d_{i}}$ of the anchor nodes and pay attention to the estimation error of the distance between the mobile nodes. Then the location error of unknown nodes is as follows:

(14) $$\begin{array}{ll} \left(\begin{array}{c} \delta \hat{x}\\ \delta \hat{y} \end{array}\right) & =\sum_{i=1}^{k}S_{d_{i}}\delta_{d_{i}}\\ & =\sum_{i=1}^{k}\frac{\partial ({\left(A^{T}A\right)}^{-1}A^{T}B)}{\partial d_{i}}\delta_{d_{i}} \end{array}$$

Then we can get the standard deviation of localization result through Equation (12) according to the square root rule:

(15) $$\begin{array}{ll} \left(\begin{array}{c} \sigma_{x}\\ \sigma_{y} \end{array}\right) & =\sqrt{\sum_{i=1}^{k}{\left(\frac{\partial ({\left(A^{T}A\right)}^{-1}A^{T}B)}{\partial d_{i}}\right)}^{2}\sigma_{d_{i}}^{2}}\\ & =\sqrt{\sum_{i=1}^{k}{\left(2{\left(A^{T}A\right)}^{-1}A^{T}\left(\begin{array}{c} 0\\ \vdots \\ 1\\ \vdots \\ 0 \end{array}\right)d_{i}\sigma_{d_{i}}\right)}^{2}} \end{array}$$

From Equation (15), we can see that the standard deviation of localization result has a direct relationship with the distance estimation results and their corresponding standard deviation information. In addition, the error of the positioning result is proportional to the standard deviation of the distance estimation $d_{i}$, and it is also proportional to the product of the estimated distance and the standard deviation of the corresponding estimation $d_{i}\cdot \sigma_{d_{i}}$(it is defined as the error propagation factor).

Therefore, Equation (15) shows that the smaller the range standard deviation and error propagation factor, the smaller the localization error. According to this relationship, we propose the MSDO criteria and MEPO criteria.

After uncertainty propagation analysis, the optimally selected anchor nodes’ ranging information will be applied to the least-squares localization method, which can effectively reduce the localization error in theory. It is mainly about the following four steps:

- The anchor nodes are accurately placed in the site with a known location, and the coordinate of anchor nodes is obtained;
- Each mobile node receives the range estimation of anchor node 150 times, in which there are k anchor nodes;
- The mean values and standard deviation of 150 ranging numbers are calculated statistically, and the standard deviation and error propagation factors are sorted from small to large, the sort order represents the quality order of nodes;
- According to the MSDO and MEPO criteria, we obtain the index of the corresponding anchor nodes (we select the nodes with index from 1 to 5). Then the selected anchor nodes and their corresponding distance estimation results are applied to the least-squares localization method, which will obtain the initial localization result.

**Figure 4:**
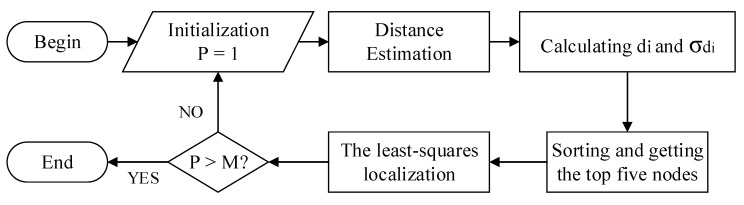
Procedures of the MSDO and MEPO localization method.

We illustrate this process in Figure 4.

### 3.4. Improvement of the Localization Results with Particle Filter Algorithm

The particle filter algorithm has outstanding advantages in solving the optimal estimation problem of the nonlinear non-Gaussian system, and it is also widely used in a nonlinear mobile positioning system.

After statistically calculating the distances estimation of the anchor nodes to the moving nodes, according to the MSDO criterion and the MEPO criterion, we bring the distance information and its coordinate information of the selected reliable anchor nodes into the least-squares localization algorithm to obtain the preliminary localization results. 

In this section, we construct the state equation and observation equation after inputting the distance estimation results of the anchor node to the initial localization coordinates. The positions information of the optimized node is obtained by particle filter algorithm to track the motion state of the moving node. The distributed localization methods based on anchor node selection and particle filter optimization have been proposed, called MSDO-PF and MEPO-PF. The following is a detailed illustration.

Suppose that the motion model of the mobile node is as follows:

(16) $$\{\begin{array}{c} X_{k}=f_{k}\left(X_{k-1},\delta_{k}\right)\\ Y_{k}=h_{k}\left(X_{k},\gamma_{k}\right) \end{array}$$

where k denotes the motion time of mobile nodes, random variable $X_{k}$ denotes the predicted value of target location, and $Y_{k}$ denotes the observed value of the target position. In this method, $Y_{k}$ is the preliminary result of positioning after the optimization of anchor nodes. $f_{k}$ and $h_{k}$ are nonlinear function. $\delta_{k}$ denotes the system noise and $\gamma_{k}$ denotes the observation noise, both are independent.

Construct a set ${\left\{X_{k}^{\left(i\right)},W_{k}^{\left(i\right)}\right\}}_{i=1}^{N}$ containing N particles, where $X_{k}^{\left(i\right)}$ represents the state of the *i*th particle at the moment, $W_{k}^{\left(i\right)}$ represents the weight of this particle, and the weights satisfied that $\sum_{i=1}^{N}W_{k}^{\left(i\right)}=1$. $f\left(X_{k}\right)$ denotes the real coordinates of the target at time *k*, and *A* is the posterior probability density of $X_{k}$ at this time. Then the final positioning result is expressed as:

(17) $$E\left[f\left(X_{k}\right)\right]=\int f\left(X_{k}\right)p\left(X_{k}|Y_{1:k}\right)dX_{k}$$

In practical application, it is challenging to extract effective samples directly from a posterior probability distribution. So Sequential Importance Sampling (SIS) is introduced to improve sampling efficiency. SIS extracts samples of the known importance sampling density $q\left(X_{k}|Y_{k}\right)$ and avoids directly extracting samples of $p\left(X_{k}|Y_{k}\right)$. The Equation (17) can be expressed as:

(18) $$\begin{array}{ll} E\left[f\left(X_{k}\right)\right] & =\int f\left(X_{k}\right)p\left(X_{k}|Y_{1:k}\right)dX_{k}\\ & =\int f\left(X_{k}\right)\frac{p\left(X_{k}|Y_{1:k}\right)}{q\left(X_{k}|Y_{1:k}\right)}q\left(X_{k}|Y_{1:k}\right)dX_{k}\\ & =\int f\left(X_{k}\right)w_{k}\left(X_{k}\right)q\left(X_{k}|Y_{1:k}\right)dX_{k} \end{array}$$

In (18),

(19) $$w\left(X_{k}\right)=\frac{p\left(X_{k}|Y_{1:k}\right)}{q\left(X_{k}|Y_{1:k}\right)}$$

The importance density function is decomposed as follows:

(20) $$q\left(X_{1:k}|Y_{1:k}\right)=q\left(X_{1:k-1}|Y_{1:k-1}\right)q\left(X_{k}|X_{1:k-1},Y_{1:k}\right)$$

According to the importance sampling theory, the appropriate importance sampling density is selected as follows:

(21) $$q\left(X_{k}|X_{1:k-1},Y_{1:k}\right)=q\left(X_{k}|X_{1:k-1},Y_{k}\right)=p\left(X_{k}|X_{1:k-1}\right)$$

The recursive form of the posterior probability density function is as follows:

(22) $$\begin{array}{ll} p\left(X_{k}|Y_{1:k}\right) & =\frac{p\left(Y_{k}|X_{1:k},Y_{1:k-1}\right)p\left(X_{1:k}|Y_{1:k-1}\right)}{p\left(Y_{k}|Y_{1:k-1}\right)}\\ & =\frac{p\left(Y_{k}|X_{k}\right)p\left(X_{k}|X_{k-1}\right)p\left(X_{1:k-1}|Y_{1:k-1}\right)}{p\left(Y_{k}|Y_{1:k-1}\right)} \end{array}$$

Then the particle weight represented by Equation (19) can be expressed as an iterative form:

(23) $$\begin{array}{c} w_{k}^{\left(i\right)}=w_{k-1}^{\left(i\right)}\frac{p\left(Y_{k}|X_{k}^{\left(i\right)}\right)p\left(X_{k}^{\left(i\right)}|X_{k-1}^{\left(i\right)}\right)}{q\left(X_{k}^{\left(i\right)}|X_{1:k-1}^{\left(i\right)},Y_{1:k}\right)}\\ =w_{k-1}^{\left(i\right)}p\left(Y_{k}|X_{k}^{\left(i\right)}\right) \end{array}$$

Formula (23) is expressed as a recursive form:

(24) $$\begin{array}{ll} w_{k}^{\left(i\right)} & =w_{k-1}^{\left(i\right)}p\left(Y_{k}|X_{k}^{\left(i\right)}\right)\\ & =w_{0}^{\left(i\right)}\prod_{n=2}^{k}p\left(Y_{n}|X_{n}^{\left(i\right)}\right)\\ & =\frac{1}{N}\prod_{n=2}^{k}p\left(Y_{n}|X_{n}^{\left(i\right)}\right) \end{array}$$

Using the Monte Carlo sampling method, the expression (17) is as follows:

(25) $$\begin{array}{ll} E\left[f\left(X_{k}\right)\right] & =\int f\left(X_{k}\right)w_{k}\left(X_{k}\right)q\left(X_{k}|Y_{1:k}\right)dX_{k}\\ & ≃\frac{1}{N}\sum_{i=1}^{N}w\left(X_{k}^{\left(i\right)}\right)f\left(X_{k}^{\left(i\right)}\right)\\ & =\frac{\sum_{i=1}^{N}w\left(X_{k}^{\left(i\right)}\right)f\left(X_{k}^{\left(i\right)}\right)}{\sum_{i=1}^{N}w\left(X_{k}^{\left(i\right)}\right)} \end{array}$$

In Equation (25), because of:

(26) $$\begin{array}{ll} 1 & =\int_{-\infty }^{+\infty }p\left(X_{k}|Y_{1:k}\right)dX_{k}\\ & =\int_{-\infty }^{+\infty }\frac{p\left(X_{k}|Y_{1:k}\right)}{q\left(X_{k}|Y_{1:k}\right)}q\left(X_{k}|Y_{1:k}\right)dX_{k}\\ & =\frac{1}{N}\sum_{i=1}^{N}w\left(X_{k}^{\left(i\right)}\right) \end{array}$$

The weight of the particles is satisfied:

(27) $$\sum_{i=1}^{N}w\left(X_{k}^{\left(i\right)}\right)\approx N$$

The weighted particle set ${\left\{X_{k}^{\left(i\right)},W_{k}^{\left(i\right)}\right\}}_{i=1}^{N}$ is used to approximate the position of the Particle filter, and the output result is:

(28) $$E\left[f\left(X_{k}\right)\right]=\sum_{i=1}^{N}W_{k}^{\left(i\right)}f\left(X_{k}\right)$$

where the particle weight is:

(29) $$W_{k}^{\left(i\right)}=\frac{w\left(X_{k}^{\left(i\right)}\right)}{\sum_{i=1}^{N}w\left(X_{k}^{\left(i\right)}\right)}$$

At this time, there is an inevitable particle degradation problem in the particle filter algorithm. With the increase of iterations, only a few particles are close to the actual samples, and the weight of most particles is minimal, which causes a waste of computing resources. According to the theory of particle filter, we add the resampling to reduce the degradation of the Particle filter.

### 3.5. Complexity Analysis

We make computing time complexity analysis of the proposed method as following. The proposed method mainly comprises the statistical calculation of distance estimation, the optimization selection of anchor nodes, the least-square localization, and the particle filtering. The complexity of the statistical calculation is O(Nk). The complexity of the optimization selection is $O(k\log_{2}^{k})$. The complexity of the least-square localization is $O(k^{2})$. The complexity of the particle filtering is $O(PSn_{x}^{2})$. Here *N* is the repeated measurement number of distance, *k* is the number of anchor nodes, *P* is the number of particles, *S* is the number of iterations, $n_{x}$ is the number of states. 

For reference, we compare related least-square localization methods. They are the randomly selected (RS) anchor nodes [6], the proposed minimum standard deviation optimization (MSDO) and the proposed minimum error propagation optimization (MEPO), the minimum standard deviation optimization with particle filter optimization (MSDO-PE), and the minimum error propagation optimization with particle filter optimization (MEPO-PE).

Based on above analysis, the complexity of RS is $O(Nk)+O(k^{2})$. The complexity of MSDO is $O(Nk)+O(k\log_{2}^{k})+O(k^{2})=O(Nk)+O(k^{2})$. The complexity of MEPO is $O(Nk)+O(k\log_{2}^{k})+O(k^{2})=O(Nk)+O(k^{2})$. The complexity of MSDO-PE is $O(Nk)+O(k\log_{2}^{k})+O(k^{2})+O(PSn_{x}^{2})=O(Nk)+O(k^{2})+O(PSn_{x}^{2})$. The complexity of MEPO-PE is $O(Nk)+O(k\log_{2}^{k})+O(k^{2})+O(PSn_{x}^{2})=O(Nk)+O(k^{2})+O(PSn_{x}^{2})$. We show the complexity of these methods in Table 1.

From Table 1, it is illustrated that with a particle filter, the complexity of the proposed method is higher than that without a particle filter. The complexity of the MSDO-PE and MEPO-PE methods is the same level. The complexity of the RS, MSDO and MEPO methods are the same level. 

## 4. Simulation and Analysis

### 4.1. Simulation Conditions

**Figure 5:**
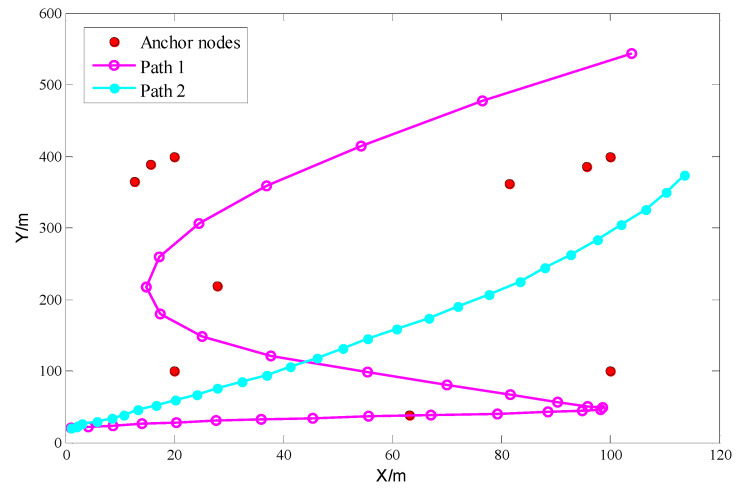
Simulation scenario.

We set a simulation scene with the size of 120 m × 600 m, as shown in Figure 5. There are four fixed nodes with coordinates (20,100), (20,400), (100,100), (100,400), respectively, and six random anchor nodes. It should be noted that there are six randomly distributed anchor nodes in each simulation experiment, which makes the experimental scene variable and verifies the applicability of the algorithm. Two nonlinear motion paths (path 1 and path 2) are set to simulate distributed motion nodes. The mobile path is sampled at an interval of one second, and 30 movement time points are selected. When arriving at the movement time points, each anchor node is selected to measure the distance of the unknown node 150 times. The mean value, standard deviation, and error propagation factor of the distance estimation of the 10 anchor nodes are calculated, respectively. In this experiment, 500 particles in the simulation scene are used. We show the experimental parameters and values in Table 2. All the experimental data are obtained in the experimental platform intel (R) i7 4720HQ@1.6 GHz, 8 GB ram, windows 10, 64 bit, Matlab 2014a.

### 4.2. Evaluation Metric

The accuracy of positioning is measured by the average positioning error $e_{MSE}$, as shown in Equation (30), which represents the absolute distance between the estimated coordinate information and the actual coordinate information of the unknown node through calculation.

(30) $$e_{MSE}=\frac{\sum_{i=1}^{k}\sqrt{{\left(x-x_{i}\right)}^{2}+{\left(y-y_{i}\right)}^{2}}}{k}$$

Among them, $\left(x_{i},y_{i}\right)$ represents the estimated position coordinates, (x,y) represents the actual position coordinates, and k represents the number of steps of mobile positioning. In addition, the smaller the average positioning error is, the higher the positioning accuracy is, and the closer the estimated position of the unknown node is to the actual position.

The Anti-interference capability of the localization system is described by error variance $e_{VAR}$, which indicates the fluctuation between the location error and the average positioning error at different time points. For different localization methods, the smaller the error variance is, the more stable the localization accuracy is, and the stronger the robustness of the system is, as shown in Equation (31):

(31) $$e_{VAR}=\frac{\sum_{i=1}^{k}{\left(e_{i}-e_{MSE}\right)}^{2}}{k}$$

where $e_{i}$ is the positioning error at the first motion time node i.

### 4.3. Localization Evaluation

#### 4.3.1. Comparison of Anchor Node Optimization Methods

**Figure 6:**
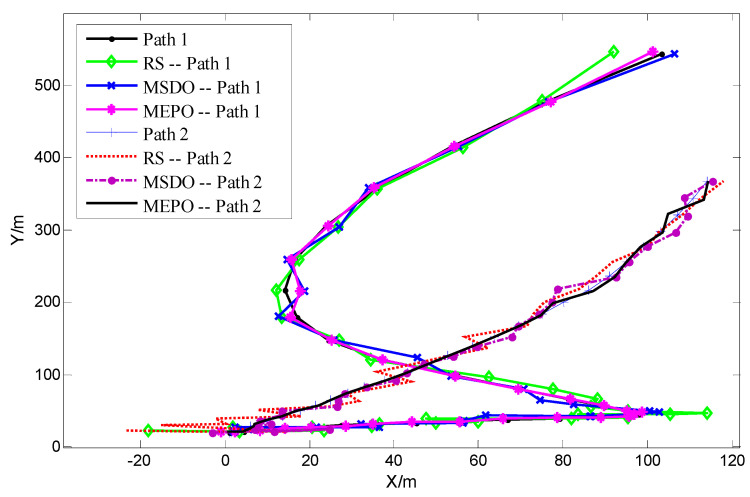
The trajectories of using RS, MSDO, MEPO node selection methods for path 1 and path 2.

In the above WSN distributed localization simulation scenario, we compare the localization results of three methods: the RS, the MSDO and the MEPO. We show the comparison results in Figure 6:

As can be seen from Figure 6, after using the MSDO and the MEPO to select the anchor nodes, the tracking trajectories of path 1 and path 2 are closer to the real trajectories than those of RS anchor nodes. This shows that our anchor node optimization algorithm can improve the localization accuracy obviously.

The experiment was repeated five times independently, and the positioning data of three anchor node selection methods were calculated. We statistically calculate the localization effects of path 1 and path 2 as shown in Table 3 and Table 4, respectively:

According to Table 3 and Table 4, for the least-squares localization method, the anchor nodes optimization based on MSDO of path 1 and path 2 can improve the location accuracy by 15.4% and 17.8%, respectively. The anchor node optimization based on MEPO of path 1 and path 2 can improve the positioning accuracy by 51.5% and 51.4%.

The error variance of path 1 and path 2 based on MSDO can be reduced by 14.4% and 15.5%. The error variance of path 1 and path 2 based on MEPO can be reduced by 76.7% and 76.1%, respectively.

The improvement of localization performance is mainly due to high-quality distance estimation and results are selected to participate in the localization calculation by MSDO or MEPO, so the localization error becomes smaller, and the tracking trajectory is close to the actual motion trajectory.

#### 4.3.2. Estimation of the Location Results with Particle Filter

**Figure 7:**
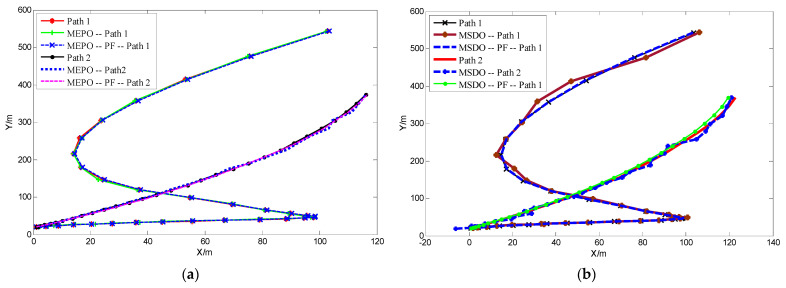
(**a**) The trajectories of MEPO localization method using PF or not (**b**) The trajectories of MSDO localization method using PF or not.

**Figure 8:**
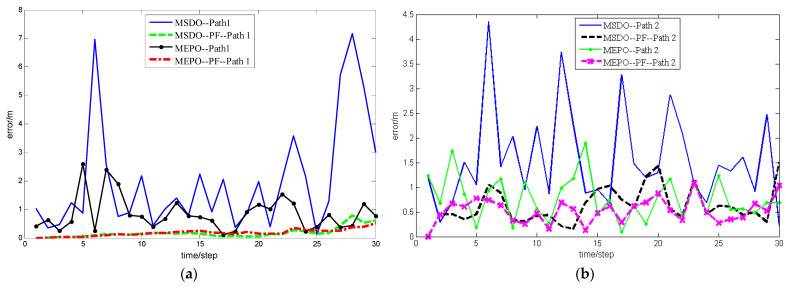
(**a**) Localization error of Path 1 using PF or not (**b**) Localization error of Path 2 using PF or not.

We use MEPO, MSDO and RS localization algorithms combined with the particle filter to optimize the localization results, the trajectory tracking results are shown in Figure 7:

When MEPO or MSDO localization method is used, it can be seen from Figure 7 that the tracking trajectory optimized by particle filter is significantly better. For the convenience of analysis, the corresponding localization error analysis diagrams are shown in Figure 8:

Figure 8 clearly show that the particle filter greatly improves the localization accuracy and system robustness when using the minimum error propagation and minimum standard deviation optimization.

To quantitatively measure the contribution of the anchor nodes optimization algorithm to the localization accuracy and system robustness under the premise of using particle filter, we independently conducted localization simulations five times. We statistically calculated the localization effects of path 1 and path 2 after using the MSDO method and particle filter (MSDO-PF) or MEPO method and particle filter (MEPO-PF), the experiment dates are as shown in Table 5, Table 6 and Table 7.

According to the statistical calculation, when the MSDO method based on particle filter is utilized to optimize the anchor node localization, compared with the RS method based on particle filter, the localization accuracy and the error variance of path 1 are equivalent. The localization accuracy of path 2 is improved by 5.9%, and the error variance is reduced by 7.8%.

When MEPO-PF is used to optimize localization, compared with RS-PF, the localization accuracy of path 1 is improved by 14.8% and the error variance is reduced by 56.3%; the localization accuracy of path 2 is the same, but the error variance is reduced by 5.5%. Based on the above quantitative analysis, it can be seen that the anchor node optimization algorithm proposed in this paper not only effectively improved positioning accuracy but also improved the robustness of the system under the premise of the same use of particle filtering.

#### 4.3.3. Localization Efficiency Evaluation

**Figure 9:**
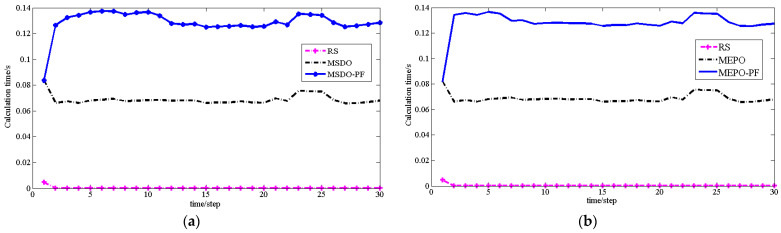
(**a**) The calculation time of MSDO using PF or not (**b**) The calculation time of MEPO using PF or not.

The combination of the particle filter algorithm has an outstanding contribution to improving the localization accuracy and enhancing the system robustness. However, there is no doubt that the particle filter algorithm will increase the operation time of each localization process. As shown in Figure 9 and Table 8, we compare the calculation times of MSDO and MEPO localization algorithms using the particle filter. 

As shown in Table 8, the time consumed by PF based positioning algorithm is much longer than that without PF. However, due to the advantages of the distributed system, each mobile node is parallel in completing the location calculation, and the total calculation time of the system will not rise with the increase of mobile nodes to be located.

## 5. Conclusions

To improve the positioning accuracy and robustness of the WSN distributed mobile localization system, this paper deduces the anchor node optimization algorithm based on minimum standard deviation and minimum error propagation by analyzing the error propagation of the range-based positioning algorithm. Through the optimization selection of anchor nodes, reliable ranging information is brought into the least-squares positioning method, and the simulation results show that the optimization of the anchor node can improve the localization accuracy effectively. Based on the introduction and analysis of the application of particle filter algorithm in positioning algorithm, we propose the distributed localization method based on anchor node selection and particle filter optimization (MSDO-PF and MEPO-PF). Through simulation and the analysis of the average localization error and error variance, it is verified that the MSDO-PF and MEPO-PF method not only improves the positioning accuracy but also has a good filtering effect on the peak error, which means the robustness of the system is improved. Finally, the localization efficiency of the optimization localization algorithm combined with particle filter is analyzed. The proposed method in this paper has a noticeable improvement in positioning accuracy and the system robustness of the WSN distributed mobile positioning system at the expense of certain time efficiency. 

## Figures and Tables

**Table 1:** Complexity analysis of the localization methods.

Methods	RS	MSDO	MEPO
Complexity	$O(Nk)+O(k^{2})$	$O(Nk)+O(k^{2})$	$O(Nk)+O(k^{2})$
**Methods**	**MSDO-PF**		**MEPO-PF**
Complexity	$O(Nk)+O(k^{2})+O(PSn_{x}^{2})$		$O(Nk)+O(k^{2})+O(PSn_{x}^{2})$

**Table 2:** Experimental parameters and values.

Parameters	Value
Scene size	120 m × 600 m
Anchor node number	10
Mobile node number	2
Fixed nodes	(20,100), (20,400), (100,100), (100,400) (m)
Random anchor nodes	randomly distributed
Simulation step	30
Ranging repeat times	150
Particle number	500

**Table 3:** Comparison of localization effects of different anchor nodes selection methods in path 1.

Methods	$\mathrm{Average}\;\mathrm{Positioning}\;\mathrm{Error}\;e_{MSE}\;(m)$	$\mathrm{Error}\;\mathrm{Variance}\;e_{VAR}$ $\;(m^{2})$
RS	2.45	4.87
MSDO	2.07	4.16
MEPO	1.18	1.13

**Table 4:** Comparison of localization effects of different anchor nodes selection methods in path 2.

Methods	$\mathrm{Average}\;\mathrm{Positioning}\;\mathrm{Error}\;e_{MSE}\;(m)$	$\mathrm{Error}\;\mathrm{Variance}\;e_{VAR}$ $\;(m^{2})$
RS	2.23	3.85
MSDO	1.83	3.25
MEPO	1.08	0.92

**Table 5:** The localization effects of using RS method and particle filter (RS-PF).

Methods	$\mathrm{Average}\;\mathrm{Positioning}\;\mathrm{Error}\;e_{MSE}\;(m)$	$\mathrm{Error}\;\mathrm{Variance}\;e_{VAR}$ $\;(m^{2})$
Path1 + PF	0.33	0.12
Path2 + PF	1.34	0.51

**Table 6:** The localization effects of using MSDO method and particle filter (MSDO-PF).

Methods	$\mathrm{Average}\;\mathrm{Positioning}\;\mathrm{Error}\;e_{MSE}\;(m)$	$\mathrm{Error}\;\mathrm{Variance}\;e_{VAR}$ $\;(m^{2})$
Path1 + PF	0.35	0.13
Path2 + PF	1.26	0.47

**Table 7:** The localization effects of using MEPO method and particle filter (MEPO-PF).

Methods	$\mathrm{Average}\;\mathrm{Positioning}\;\mathrm{Error}\;e_{MSE}\;(m)$	$\mathrm{Error}\;\mathrm{Variance}\;e_{VAR}$ $\;(m^{2})$
Path1 + PF	0.28	0.05
Path2 + PF	0.05	0.48

**Table 8:** Comparison of the positioning simulation time.

Methods	RS	MSDO	MEPO	MSDO-PF	MEPO-PF
Time(s)	0.0002	0.0687	0.0688	0.1286	0.1279

## Data Availability

Not applicable.
